# Nonlocal single particle steering generated through single particle entanglement

**DOI:** 10.1038/s41598-021-85508-8

**Published:** 2021-03-24

**Authors:** L. M. Arévalo Aguilar

**Affiliations:** Facultad de Ciencias Físico Matemáticas, Benemérita Universidad Autónoma de Puebla, 18 Sur y Avenida San Claudio, Col. San Manuel, C.P. 72520 Puebla, PUE Mexico

**Keywords:** Information theory and computation, Quantum physics

## Abstract

In 1927, at the Solvay conference, Einstein posed a thought experiment with the primary intention of showing the incompleteness of quantum mechanics; to prove it, he employed the instantaneous nonlocal effects caused by the collapse of the wavefunction of a single particle—*the spooky action at a distance*–, when a measurement is done. This historical event preceded the well-know Einstein–Podolsk–Rosen criticism over the incompleteness of quantum mechanics. Here, by using the Stern–Gerlach experiment, we demonstrate how the instantaneous nonlocal feature of the collapse of the wavefunction together with the single-particle entanglement can be used to produce the nonlocal effect of steering, i.e. the single-particle steering. In the steering process Bob gets a quantum state depending on which observable Alice decides to measure. To accomplish this, we fully exploit the spreading (over large distances) of the entangled wavefunction of the single-particle. In particular, we demonstrate that the nonlocality of the single-particle entangled state allows the particle to “know” about the kind of detector Alice is using to steer Bob’s state. Therefore, notwithstanding strong counterarguments, we prove that the single-particle entanglement gives rise to truly nonlocal effects at two faraway places. This opens the possibility of using the single-particle entanglement for implementing truly nonlocal task.

## Introduction

Einstein efforts to cope with the challenge of the conceptual understanding of quantum mechanics has been a source of inspiration for its development; in particular, the fact that the quantum description of physical reality is not compatible with causal locality was critically analized by him. In 1927 Einstein posed a simple thought experiment where a *single-particle* experiencing diffraction by a single slit—hence generating an expanding spherical wavefunction—reaches a screen; as a result, “*the particle must be considered as potentially present with almost constant probability over the whole area of the screen; however, as soon as it is localized, a peculiar action-at-a-distance must be assumed to take place which prevents the continuously distributed wave in space from producing an effect at two places on the screen*”^1–3^. That is to say, the wavefunction must collapse at the point where the particle is detected, hence, different points situated far away must instantaneously be unable to detect the particle. Notice that it was at the Solvay conference where, as far as we know, the phrase “a peculiar action-at-a-distance” was used for the first time by Einstein but in the context of a single-particle only, besides it is important to notice that this phrase has not been used in the Einstein-Podolsky-Rosen analysis. Additionally, in a letter written in 1947 to Born, Einstein used the phrase *spooky action at a distance*^4^. Hence, it would be worth quoting Einstein’s words: “*...I admit, of course, that there is a considerable amount of validity in the statistical approach...I cannot seriously believe in it because the theory cannot be reconciled with the idea that physics should represent a reality in time and space free from spooky actions at a distance*^4^,” in this sentence Einstein talk about whether or not the wavefunction describes the Born probability for a single particle, i.e. the phrase *spooky actions at a distance* refers also to a single particle. Then, historically, by using this thought experiment Einstein was able to put the nonlocal effects of quantum mechanics and the collapse of the wavefunction as a distinctness of quantum mechanics that should be investigated. Some years later from the Solvay coference, in a letter to Schrödinger written in 1935, Einstein reframe this thought experiment in terms of boxes, although as a classical analogy only^3^.

**Figure 1:**
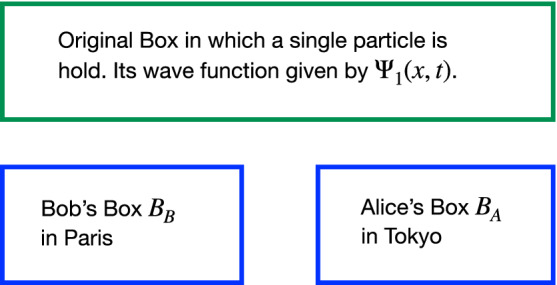
A single particle with wavefunction given by $\Psi _1(x,t)$ is hold in the original Box. After that, the original Box is divide in two Boxes, one of them carried to Paris by Bob and the other one carried to Tokyo by Alice, therefore the wavefunction after the splitting is given by $\Psi _2=\phi _{Paris}(x)+\phi _{Tokyo}(x)$.

Meanwhile, de Broglie gave his own version for this though experiment in 1962^3^, see also refernce^5^, by using the box thought experiment in which a single particle was situated and where its wave function was given by $\Psi _1(x,t)$; afterwards the box is divided into two boxes, one of them carried to Paris, by Bob, say. The other one carried by Alice to Tokyo. Hence after this division the wavefunction is given by $\Psi _2=\phi _{Paris}(x)+\phi _{Tokyo}(x)$, see Fig. 1. Consequently, if Bob opened his box in Paris and found the particle in it, the wave function would collapses to $\Psi _f=\phi _{Paris}(x)$. Besides, de Broglie call attention to the astonishing nonlocal effect of Alice opening her box in Tokyo and finding *nothing* inside, but nevertheless producing the *collapse* of the wavefunction; in this case the wavefunction $\Psi _2=\phi _{Paris}(x)+\phi _{Tokyo}(x)$ also collapses to $\Psi _f=\phi _{Paris}(x)$ in Paris.

The Einstein’s boxes resemble the nolocality of single photons, first addressed by Tan et al.^6^ and further used by Hardy to rule out local hidden variables^7^, see also the work of Peres^8^. The nonlocality of single photons has raised great debate which includes their experimental demonstration^9^, new theoretical and experimental proposal to test it^10–14^ and criticism^15–17^. It is worth mentioning that the nonlocal character of the collapse of the wavefunction was experimentally demonstrated by Fuwa et al.^18^, see also references^19–21^. In reference^20^ an experiment testing the collapsing time of a single-photon state was carried out and in reference^21^ an experimental demonstration of the Einstein’s thought experiment of 1927 at the Solvay conference was demonstrated. The single-photon steering was experimentally demonstrated in a detection loophole free scenario by Guerreiro et al.^22^, the name single-photon steering was stated by N. Brunner^23^.

In the same spirit, the nonlocality phenomenon was also considered in the single-particle entanglement (or intraparticle entanglement), where the entanglement occurs with at least two degree of freedom of a single particle^24^. It has been addressed, for example, with electrons^25^. However, the single-particle entanglement faced critical comments over its possible nonlocality properties^17,26,27^; this criticism claims that the entanglement of a single-particle is contextual only, denying nonlocal effects in it. Interestingly, in some of this works the Einstein’s phrase over the peculiar action-at-a-distance is only used for the multi-particle entanglement case and ruling out its applicability to the sinlge-particle entanglement situation^26–29^; however, as it was already stated, the first time that Einstein himself used that phrase was for the single-particle case and without considering entanglement. Here, we shown that the single-particle entanglement in the SGE possesses truly nonlocal properties by showing how Alice can steer Bob’s states.

On the other hand, before Einstein raised his doubts at the Solvay conference held in 1927, in 1921 Stern proposed an experiment with the aim of testing the Bohr quantization rule of the orbital angular momentum^30^. Stern conducted that experiment in 1922 with the help of Gerlach. Meanwhile, in 1925 Uhlenbeck and Goudsmit proposed the idea of the existence of the internal spin to explain the fine structure phenomena in the espectral emission^31^. However, it was until 1927 that the scientific community began to realize that what the Stern–Gerlach experiment (SGE) really proved was the existence of the internal spin^31^. From then on, the SGE has been a fundamental tool for the development of quantum mechanics; which, as it was explained in references^32,33^, it is an entanglement device. In fact, the quantum attributes of the SGE and a new explanation for how it works and has been given in many papers, see for example reference^34^; in particular, in references^32,33,35^ the Schrödinger equation for this experiment has been solved and the violation of local realism was proved in reference^36^. It is well worth mentioning that recent experimental evidence confirms the existence of the superposition of the wavepackets^37,38^. As it was stated in the previous paragraph, in this work we demonstrated that the quantum properties of the SGE together with the nonlocality of the wavefunction can be used to tailor the steering effect, a process which we can call *single-particle steering*. Steering is a different nonlocal property of quantum mechanics that is stronger that noneparability and weaker that Bell nonlocality^39–45^, which allow Alice to realise many nonclassical task like steering nonclassicality, where she steer Bob’s state into a nonclassical state, this process was named *nonclassical steering *^46^. Nonlocality depends on the uncertainty principle^47^ and the steering^48–50^.

## The physics of the Stern–Gerlach experiment

Here, we analyze how the Stern–Gerlach experiment works in a quantum mechanical way when we send individual atoms one by one. As it was demonstrated in references^32,33^ the evolution of the wave function in the Stern–Gerlach experiment is given by:

1 $$\begin{aligned} |{\psi (t)}\rangle= & {} C_0M(x,y) \times \Big \{ e^{\frac{-it\mu _c}{\hbar }(B_{0}+bz)} \exp \left[ \frac{-1}{4(\sigma _{0}^{2}+i t\hbar /2m)}\left( z+\frac{t^{2}\mu _c b}{2m}\right) ^{2}\right] |{\uparrow _z}\rangle \nonumber \\&+\, e^{\frac{i t\mu _c}{\hbar }(B_{0}+bz)} \exp \left[ \frac{-1}{4(\sigma _{0}^{2}+i t\hbar /2m)}\left( z-\frac{t^{2}\mu _c b}{2m}\right) ^{2}\right] |{\downarrow _z}\rangle \Big \}, \end{aligned}$$

where

2 $$\begin{aligned} C_0=e^{\frac{-it^3\mu _c^2b^2}{6m\hbar }}\frac{1}{\sqrt{2}} \left[ \frac{\sigma _{0}}{(2\pi )^{1/2}}\right] ^{3/2} \left( \sigma _{0}^{2}+\frac{i \hbar t}{2m}\right) ^{-3/2}, \end{aligned}$$

and

3 $$\begin{aligned} M(x,y)=e^{-\sigma _0^2k_y^2} e^{\frac{ 4y\sigma _{0}^{2}k_{y}}{4(\sigma _{0}^{2}+ t\hbar /2m)}} \exp \left[ \frac{-(x^{2}+y^{2}-4\sigma _{0}^{4}k_{y}^{2})}{4(\sigma _{0}^{2}+ it\hbar /2m)}\right] . \end{aligned}$$

Now, in order to analize in detail the physics given by this equation: First, notice that what Eq. (1) tell us is that the SGE is an entanglement device, contrary to the usual understanding that considers the SGE as a spin measurement device. That is, there is not any wavefunction collapse in the SGE as it is required by the collapse postulate of quantum mechanics when a measurement is done (Fifth Postulate in reference^51^), instead what the SGE produces is the entangled state given by Eq. (1). In fact, it was shown that the entangled state given by Eq. (1) violates the Bell’s Clauser–Horne–Shimony–Holt kind inequality^36^.

Second, the wavefunction given in Eq. (1) consists of two Gaussian functions—that are entangled with the spin of the particle—which are separating one from another in the *z* axe; at time *t* the Gaussians are centred, respectively, at position $z=\pm \frac{t^2\mu _c b}{2m}$ , and they are moving at a speed $\frac{\mu _c bt}{2m}$ in opposite directions, which in turn imply a constant acceleration of $\frac{\mu _c b}{2m}$. Consequently, these Gaussians represent the movement of the external degrees of freedom and correspond to the possible values that these degree of freedom get when measurements are done. To see a recent demonstration that in quantum mechanics the first Newton law is ruled out see the work of Hofmann^52^.

### Measuring the position observable

If a screen is placed for measuring the possible positions (nowadays an alternative is a device which measure the position by sensing the cloud of electrons that is released when the particle hit it, see Arévalo Aguilar, On the Stern–Gerlach experiment, to be published) in the *z* axe, as it was done in the original experiment, two different spots are got. We recall that there exist a connection between physical properties and measurements formulated in the form of a self-adjoint operator^53^; this connection is realized in the combination of the eigenstate of the operator with a corresponding eigenvalue^53^. Hence, taking into account that the *z* position is being measured, then the wavefunction given by Eq. (1) collapses (with 50% of probability) towards the eigenstate associated with the eigenvalue of the operator *z* that was obtained:

4 $$\begin{aligned} |{\psi (t)}\rangle =C_0^1M(x,y) |{ Z_+}\rangle |{\uparrow _z}\rangle , \end{aligned}$$

for the upper spot, where $|{Z_+}\rangle$ is an eigenket of the operator *z*, and $\langle {z}|{Z_+}\rangle =\delta \left( z+\frac{t^{2}\mu _c b}{2m}\right)$ is the Dirac delta function, i.e. the wavefunction in *z* collapses to a well definite position at $z\approx \frac{t^{2}\mu _c b}{2m}$ and $C_0^i$ is normalization coefficient derived from $C_0$, here $i=1$ and below $i=2,3,4,5,6,7,8$. Notice that this spot is still uncertain about the *x* and *y* positions and about the momentum $p_z$, in fact the uncertainty in $p_z$ is maximun. The wavefunction given by Eq. (4) possesses a definite spin $|{\uparrow _z}\rangle$ and definite position in *z*. However, notice that in this case we are measuring the *z* position, not the spin, and by detecting the particle at the position eigenstate $\langle {z}|{Z_+}\rangle =\delta \left( z+\frac{t^{2}\mu _c b}{2m}\right)$ we can infer the spin value and the collapsed spin state, i.e. $\hbar /2$ and $|{\uparrow _z}\rangle$ respectively.

At the lower spot the wave function collapses to:

5 $$\begin{aligned} |{\psi (t)}\rangle =C_0^2M(x,y) |{ Z_-}\rangle |{\downarrow _z}\rangle , \end{aligned}$$

where $|{Z_-}\rangle$ is an eigenket of the operator *z*, and $\langle {z}|{Z_-}\rangle =\delta \left( z-\frac{t^{2}\mu _c b}{2m}\right)$.

### Measuring the spin observable

On the other hand suppose that Alice wants to measure the spin degree of freedom, by using a device that measure the spin (in other words, suppose that a device that measure the spin exists), then the wavefunction given by Eq. (1) collapses (with 50% of probability) towards the eigenstate associated with the value of the obtained eigenvalue (i.e. $+ \hbar /2$) of the operator $\hat{\sigma _z}$:

6 $$\begin{aligned} |{\psi _{\sigma _{1|1}}(t)}\rangle =C_0^3M(x,y) e^{\frac{-it\mu _c}{\hbar }(B_{0}+bz)} e^{\frac{-1}{4(\sigma _{0}^{2}+i t\hbar /2m)}\left( z+\frac{t^{2}\mu _c b}{2m}\right) ^{2} } |{\uparrow _z}\rangle . \end{aligned}$$

It is important to highlight that according to the measurement postulate of quantum mechanics what really collapses is the wavefunction associated with the spin degree of freedom (which is what Alice is really measuring), the external degree of freedom in *z* still is a Gaussian. Also, notice that this wavefunction is still uncertain about the *x*, *y* and *z* positions and about the momentum $p_z$. Hence, this spin’s measurement is quite different to measure the position as it was done in Eqs. (4) and (5). It is worth mentioning that this gives additional evidence to support the statement that the Stern–Gerlach apparatus is not a measurement device, but it is an entanglement device instead.

Similar considerations apply if the eigenvalue $-\hbar /2$ is obtained after the measurement of the spin degree of freedom. In this case, the collapsed wavefunction will be given by:

7 $$\begin{aligned} |{\psi _{\sigma _{2|1}}(t)}\rangle =C_0^4M(x,y) e^{\frac{it\mu _c}{\hbar }(B_{0}+bz)} e^{\frac{-1}{4(\sigma _{0}^{2}+i t\hbar /2m)}\left( z-\frac{t^{2}\mu _c b}{2m}\right) ^{2} } |{\downarrow _z}\rangle . \end{aligned}$$

## Eintein’s boxes with single-particle entanglement

The de Broglie’s version of the Einstein boxes was given in terms of one particle without single-particle entanglement. Consider now the same thought experiment but now think over that during the splitting process the particle develops single-particle entanglement between the internal and the external degree of freedom. In mathematics: suppose that the initial state of the original box is given by $|{\Psi _1(x)}\rangle =c_0|{\varphi }\rangle \left( |{\uparrow }\rangle +|{\downarrow }\rangle \right)$ where $\langle {x}|{\varphi }\rangle =\varphi (x)$ is the *x* representation of the external degree of freedom, $|{\uparrow }\rangle$ and $|{\downarrow }\rangle$ are the internal spin and $c_0$ is a constant. Hence, after the original box’s division into two boxes, one in Paris with Bob and the other one in Tokyo with Alice, the wavefunction is given by $|{\Psi _2}\rangle =c_0\left( |{\varphi _{Paris}}\rangle |{\uparrow }\rangle +|{\varphi _{Tokyo}}\rangle |{\downarrow }\rangle \right)$. Consequently, the wavefucntion will collapse depending on what observable Alice decided to measure. For example, if Alice decided to measure the spin observable (and got the value $-\hbar /2$), then the wave function would collapse to $|{\varphi _{Tokyo}}\rangle |{\downarrow }\rangle$, where $\langle {x}|{\varphi _{Tokyo}}\rangle =\varphi _{Tokyo}(x)$. On the other hand, if she decided to measure the position observable (and she found the particle at Tokyo), then the wavefunction would collapse to $|{\delta _{Tokyo}}\rangle |{\downarrow }\rangle$, where $\langle {x}|{\delta _{tokyo}}\rangle =\delta (x-x_{Tokyo})$ is the Dirac delta function in *x*.

**Figure 2:**
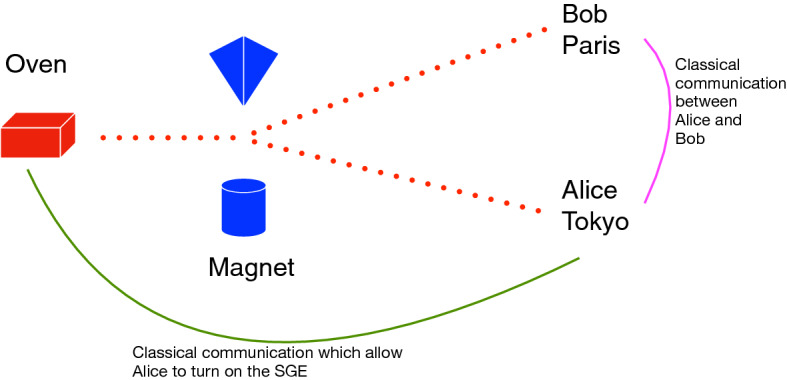
SGE featuring the Einstein’s boxes. Where the red box is the oven, the blue box represents the magnet, the red dot represents the fact that there is not classical trajectories, see references^32,33^. Alice could communicate with Bob by using the classical channel in magenta. Moreover, Alice is in full control of the SGE by using the classical channel in green, and she possess the ability to turn it on and to chose between a single or *N* atoms.

Additionally, in the case of the SGE, suppose that just one atom is sent by Alice, and that Alice is in Tokyo and Bob is in Paris. Also, suppose that Alice has automatized the SGE (which is located at suitable place) in such a way that she is able to turn it on (and with the ability to choice whether to send a single atom or *N* atoms one by one) sending a classical communication, as shown in Fig. 2.

The sep-up of Fig. 2 is a scheme of the Einstein’s boxes with single-particle entanglement; to perceive this, notice that:

1. If Alice measured the position then (we would recall that Alice is sending a single particle), if her position measuring device registered the particle’s position, then the eigenfunction of the particle would collapse to the one given by Eq. (5), i.e. $\ |{\psi (t)}\rangle =C_0^2M(x,y) |{ Z_-}\rangle |{\downarrow _z}\rangle$.
2. However, if her position measuring device registered nothing, then the eigenfunction of the particle would collapse—at Bob’s location in Paris—to the one given by Eq. (4), i.e. $|{\psi (t)}\rangle =C_0^1M(x,y) |{ Z_+}\rangle |{\uparrow _z}\rangle$.
3. On the other hand, when Alice decide to measure the spin ${\hat{\sigma }}_z$, we have: If her spin measuring device registered the eigenvalue $-\hbar /2$, then the wavefunction would collapses towards Eq. (7), i.e. $|{\psi _{\sigma _{2|1}}(t)}\rangle =C_0^4M(x,y) e^{\frac{it\mu _c}{\hbar }(B_{0}+bz)} e^{\frac{-1}{4(\sigma _{0}^{2}+i t\hbar /2m)}\left( z-\frac{t^{2}\mu _c b}{2m}\right) ^{2} } |{\downarrow _z}\rangle$ at her place.
4. However, if her spin measurement device registered nothing, then the wavefunction would collapses—at Bob’s location in Paris—towards Eq. (6), i.e.
  $$ |{\psi _{\sigma _{1|1}}(t)}\rangle =C_0^3M(x,y) e^{\frac{-it\mu _c}{\hbar }(B_{0}+bz)} e^{\frac{-1}{4(\sigma _{0}^{2}+i t\hbar /2m)}\left( z+\frac{t^{2}\mu _c b}{2m}\right) ^{2} } |{\uparrow _z}\rangle $$.

### Still there are more alternatives: different spin basis

If Alice decided to measure in a different spin basis, for example ${\hat{\sigma }}_x$, she could be able to steer a different state. In this case, rewriting Eq. (18) in the ${\hat{\sigma }}_x$ basis, we have:

8 $$\begin{aligned} |{\psi (t)}\rangle =C_0 M(x,y)\Big \{ \left[ \langle {z}|{\varphi _+}\rangle +\langle {z}|{\varphi _-}\rangle \right] |{\uparrow _x}\rangle + \left[ \langle {z}|{\varphi _+}\rangle -\langle {z}|{\varphi _-}\rangle \right] |{\downarrow _x}\rangle \Big \} \end{aligned}$$

where

9 $$\begin{aligned} \langle {z}|{\varphi _+}\rangle =e^{\frac{-it\mu _c}{\hbar }(B_{0}+bz)} e^{\left[ \frac{-1}{4(\sigma _{0}^{2}+i t\hbar /2m)}\left( z+\frac{t^{2}\mu _c b}{2m}\right) ^{2}\right] }, \end{aligned}$$

and

10 $$\begin{aligned} \langle {z}|{\varphi _-}\rangle =e^{\frac{it\mu _c}{\hbar }(B_{0}+bz)} e^{\left[ \frac{-1}{4(\sigma _{0}^{2}+i t\hbar /2m)}\left( z-\frac{t^{2}\mu _c b}{2m}\right) ^{2}\right] }. \end{aligned}$$

Therefore the following alternatives arise:

1. If her spin ${\hat{\sigma }}_x$ measuring device registered the eigenvalue $\hbar /2$, then the wavefunction would collapse towards
  11 $$\begin{aligned} |{\psi _{\sigma _{1|2}}(t)}\rangle =C_0^5M(x,y) \left[ \langle {z}|{\varphi _+}\rangle +\langle {z}|{\varphi _-}\rangle \right] |{\uparrow _x}\rangle , \end{aligned}$$
  where $\left[ \langle {z}|{\varphi _+}\rangle +\langle {z}|{\varphi _-}\rangle \right]$ is a superposition state of “being” in Paris and Tokyo at the same time.
2. If her spin ${\hat{\sigma }}_x$ measuring device registered the eigenvalue $-\hbar /2$, then the wavefunction would collapse towards
  12 $$\begin{aligned} |{\psi _{\sigma _{2|2}}(t)}\rangle =C_0^6M(x,y) \left[ \langle {z}|{\varphi _+}\rangle -\langle {z}|{\varphi _-}\rangle \right] |{\downarrow _x}\rangle , \end{aligned}$$
  where $\left[ \langle {z}|{\varphi _+}\rangle -\langle {z}|{\varphi _-}\rangle \right]$ is a superposition state of “being” in Paris and Tokyo at the same time.

### Still there are more alternatives: momentum

If Alice decided to measure the momentum ${\hat{p}}_z$, then the wave function would collapse to the eigenfunction associated with the eigenvalue ($-p_z$) of ${\hat{p}}_z$ obtained at her momentum measuring device in Tokyo (with the associated spin $|{\downarrow _z}\rangle$); but if her momentum measuring device detected noting, then the wavefunction would collapse, at a different location—i.e. at Bob’s place in Paris-, towards an eigenfunction of the momentum, i.e. the one associated with the eigenvalue $+p_z$ (with the associated spin $|{\uparrow _z}\rangle$).

## About measurement and nonlocalilty

In the previous subsections we analysed the nonlocal effects from the point of view of steering, i.e. measurements that produce assemblages like $\sigma _{a|x}=tr_A\{ \rho _{AB}(A_{a|x}\otimes {\mathbb {I}})\}$^54,55^. As it has been proved, this approach establishes a one to one relation between steering and joint measurability^54,55^; that is to say, a set of observables is not jointly measurable only if they are useful to produce steering. In this case, Alice can demonstrate entanglement by measuring two incompatible operators, for example ${\hat{\sigma }}_z$ and ${\hat{\sigma }}_x$. Notice that in the original EPR argument the entangled wavefunction was given by $\psi (x_1,x_2)=\int _{-\infty }^\infty \psi _{p}(x_2) u_p(x_1)dp$, where, according to EPR, the wavefunction $\psi _{p}(x_2)=e^{-\frac{ ip}{\hbar }(x_2-x_0)}$ is an eigen-function of the observable ${\hat{p}}_2$ with eigenvalue $-p$, whereas $u_{p}(x_1)=e^{\frac{ ip}{\hbar }x_1}$ is an eigen-function of the observable ${\hat{p}}_1$ with eigen-value *p*, see reference^56^ for details; hence, the wavefunction $\psi (x_1,x_2)$ represent an entangled state between eigen-functions of the momentum observables ${\hat{p}}_1$ and ${\hat{p}}_2$. In the Bohm’s spin 1/2 representation of the EPR argument, the entangled wavefunction is $|{\psi }\rangle _{1/2}=(|{\downarrow _z}\rangle _1|{\uparrow _z}\rangle _2-|{\uparrow _z}\rangle _1|{\downarrow _z}\rangle _2)/\sqrt{2}$, where$|{\downarrow _z}\rangle _1$ is an eigen-function of the ${\hat{\sigma }}_z^1$ operator of the observer 1, and $|{\uparrow _z}\rangle _2$ is an eigen-function of the ${\hat{\sigma }}_z^2$ operator of the observer 2; hence, $|{\psi }\rangle _{1/2}$ represents and entangled state between the eigen-functions of the spin observables ${\hat{\sigma }}_z^1$ and ${\hat{\sigma }}_z^2$. It is worthy of mention that in both cases the entanglement occurs between eigenfunctions of operators, i.e. these eigenfunctions have associated a precise eigenvalue in all cases.

On the other hand, the state given by Eq. (1) is a hybrid entangled state^57^ where the entanglement is between a continuous variable *z* and the quantized spin, i.e. position-spin entanglement; however, the wavefuntion in *z* is not an eigenfunction of the position operator neither of the momentum operator. It produces steering by measuring the position *z* whereby Alice steer Bob’s state towards the one given by Eq. (5), i.e. $\ |{\psi (t)}\rangle =C_0^2M(x,y) |{ Z_-}\rangle |{\uparrow _z}\rangle$ or, additionally she could also measure the spin ${\hat{\sigma }}_z$ and whereby she can steer Bob’s state towards Eq. (6), i.e. $|{\psi _{\sigma _{1|1}}(t)}\rangle =C_0^3M(x,y) e^{\frac{-it\mu _c}{\hbar }(B_{0}+bz)} e^{\frac{-1}{4(\sigma _{0}^{2}+i t\hbar /2m)}\left( z+\frac{t^{2}\mu _c b}{2m}\right) ^{2} } |{\uparrow _z}\rangle$. The peculiarity of this situation is that Eq. (5) and Eq. (6) are two different states that are produced by measuring two seemingly compatible operators, of course this does not contradict the results given in references^54,55^. In this case, we conjecture that for the case of hybrid entanglement the steering could be demonstrated by measuring not just incompatible observables but also the observables associated with the two wavefunctions of the composite entangled state. In the next section, a partial positive answer is given to this conjecture by showing that there is a violation of a steering inequality formed by the averages of the operators related to the position and the spin observables in the SGE. We recall that a counter example showing that not always incompatibility imply nonlocality was given^58^, here we show that there exist process where measurement of seemingly compatible observables produce steering. Hence, in the scenario of the SGE there is a rich situation in which one observer can choose between measuring incompatible observables like ${\hat{\sigma }}_z$ and ${\hat{\sigma }}_x$, or the position *z* and the momentum $p_z$, or well a combination of them like ${\hat{\sigma }}_z$ and $p_z$.

Furthermore, at the same time, it raises the question about how the spin measurement could be conducted independent of the position measurement; as it was mentioned before, the position measurement can be carried out by detecting a cloud of electrons. Unfortunately, as fas as we known, a method for detecting the spin without inferring it by the measurement of the position has not been found, that is to say it seems that we still must infer the spin value of a particle from inference of the position’s (or momentum) measurement althought a proposal has been made for a quantum simultaneous measurement of the spin direction using an Arthurs–Kelly model, i.e. by simultaneous measurements of $\hat{\sigma}_x, \hat{\sigma}_y~\text{and}~\hat{\sigma}_z$ without measuring the particle’s position. However, its implementation must still be analysed and checked further in order to asses its probable applicability to the SGE for the quantum measurement of the spin direction of ${\hat{\sigma }}_z$ only^77^; thereby, by treating the internal and external DOF as quantum variables in the SGE, it becomes clear that there are instances where the resultant final state is not the same when measuring an observable than to infer its value from measuring other observable. There is another perspective on this analysis that considers the situation when the measurement is carried out at different places by different parties^59^. Furthermore, an additional issue arises when considering whether or not the Alice’s apparatus is close to the particle or not; in our general discussion we implicitly take the point of view that she does not have any difficulty in placing the particle in her measuring apparatus, in the situation when she would get nothing the particle will appears at the place in Paris where Bob is. However, in a real experiment a careful approach must be taken into consideration to secure the right setup of the experiment.

Additionally, a parallel consideration could be made by taking into account the nonlocal measurements of the observables, for example $\sigma _z^A\otimes \sigma _z^B$ at the same time (where $\sigma _z^A$ is an Alice operator and $\sigma _z^B$ is a Bob operator) on two subsystems. Please notice that the considerations in the previous paragraph come by locally measuring two operators, for example ${\hat{\sigma }}_z$ and ${\hat{\sigma }}_x$. At first glance, it seems that measuring two operators locally is the same than measuring them in a nonlocal way; however, although a product operator is composed of two local operators, it differs from performing two local measurements individually, especially when the subsystems are distant from one another^60,61^. In others words, by measuring the product of two operators we can induce non-local properties^62–64^, as the failure of the product rule^64^. In the case of the SGE, this parallel analysis requires further research.

As a conclusion of this subsection, it is worthy of mention that further investigation is necessary to clarify how the spin and momentum measurements could be carried out independent of the position of the particle, as well as further analysis is required to clarify the steering process which occurs when measuring two observables as ${\hat{\sigma }}_z$ and *z* or ${\hat{\sigma }}_z$ and $p_z$.

## Steering certification

According to Uola et al.^42^ there are mainly three ways to verify steering. One is through the calculation of expectation values of the form $<{\hat{A}}{\hat{B}}>$ and the demonstration that these correlations can prove steerability, usually by the violation of some steering inequality^39^. A second way is to enquire whether Bob’s assemblage ${\hat{\rho }}_{a|x}$ could be explained by means of a Local Hidden State (LHS) model; and a third way is to consider a quantum state ${\hat{\rho }}_{12}$ and checking whether this state allows to see steering when Alice makes appropriate measurements; part of this latter way for proving steering was implemented in the previous sections, where it was shown the phenomenon of steering in the SGE through suitable choice of Alice measurements. According to E. G. Calvacanti et al.^39^, the entangled states, the steerable states and the Bell-nonlocal sates are all equivalent classes for pure states^39,40^. Additionally, there is a proposal to certify steering for single systems but considering measurements at the same location, i.e. without considering space- like separation^65^. In this section, the violation of a steering inequality that proves the inadequacy of LHS model in the SGE will be shown, but first the formal definition of steering will be given, see reference^39–41^.

The steering scenario considers that one party (Bob) is capable of carrying out trusted quantum measurements whereas the other party (Alice) can not trust her own measurement devices. Let the set of all observables of Alice’s system, which belongs to its Hilbert space, be denoted by ${\mathfrak {D}}_{\alpha }$ . An element of ${\mathfrak {D}}_{\alpha }$ is denoted by *A* whereas the set of its eigenvalues $\{a\}$ is denoted by $\lambda (A)$. *P*(*a*|*A*; *W*) will denote the probability that Alice gets the result *a* when she measures *A* on a system with density matrix *W*. The measurements that Alice could perform are represented by the set ${\mathfrak {M}}_{\alpha }\subseteq {\mathcal {D}}_{\alpha }$, this set is her measurement strategy, a similar notation is for Bob’s system. Additionally, let $\rho _{\xi }$ be a preexisting local hidden state (LHS). Hence, Alice can produce the steering phenomenon by using her measurement strategy ${\mathfrak {M}}_{\alpha }$ on the state *W*, if and only if (iff) it is not the case that for all $a\in \lambda (A)$, $b\in \lambda (B)$, for all $A\in {\mathfrak {M}}_{\alpha }$, $B\in {\mathfrak {D}}_{\beta }$, we have

13 $$\begin{aligned} P(a,b|A,B,W)=\sum _\xi {\mathfrak {P}}(a|A,\xi )P(b|B;\rho _\xi ){\mathfrak {P}}_\xi \end{aligned}$$

where ${\mathfrak {P}}(a|A,\xi )$ and ${\mathfrak {P}}_\xi$ are probability distributions over the hidden variable; and $P(b|B;\rho _\xi )$ is a probability distribution compatible with quantum states, i.e. it denotes the quantum probability for obtaining the eigenvalue *b* when measuring the observable *B* in the state $\rho _\xi$. Eq. (13) establishes that the phenomenon of steering could not be produced when the joint probability of Alice and Bob’s measurements can be explained employing a LHS model for Bob and a local hidden variable (LHV) model for Alice correlated with Bob’s state^41^. Hence, the state *W* is steerable iff there exists a measurement strategy ${\mathfrak {M}}_{\alpha }$ that exhibits steering, for details see^40,41^.

Additionally, the concept of EPR-steering inequalities was introduced by E. G. Calvacanti *et al.*^39^ as a criterion for certifying steering, i.e. a violation of any of these inequalities certifies that the correlations observed by two parties cannot be explained by a local hidden state model. These authors follow in close analogy the theories of certification for entanglement and nonlocality criteria^66–69^. Furthermore, E. G. Cavalcanti et al. proved an analog of the Clauser-Horne-Shimony-Holt (CHSH) inequality for steering^70^, which is a necessary and sufficient condition for certifying steering, i.e. the violation of this inequality means that the quantum state is steerable, it is given by the following equation:

14 $$\begin{aligned} {} & \sqrt{\langle {AB+A'B}\rangle ^2+\langle {AB'+A'B'}\rangle ^2}\nonumber \\&\quad \sqrt{\langle {AB-A'B}\rangle ^2+\langle {AB'-A'B'}\rangle ^2}\le 2. \end{aligned}$$

The inequality given by Eq. (14) was demonstrated taking into account the correlations which have a LHV-LHS model between two dichotomic measurements (that form a convex set)^70^, it was also proved that it obeys an optimal upper bound equal to the Cirel’son quantity^71^, i.e. $2\sqrt{2}$. Additionally, the work of Girdhar and Cavalcanty strongly suggest that *all two-qubits states that are EPR steerable with CHSH-type correlations are also Bell nonlocal* -including mixed states-^72^ (as it was mentioned above, for pure states nonlocality, steering and nonseparability are equivalent classes); that is to say, a quantum state violates a CHSH-type steering inequality iff it violates the CHSH inequality also, *possibly for different sets of measurements*^72^. Furthermore, a similar result was reported by Costa and Angelo^73^, when searching for the maximal amount in which some of the steering inequalities are violated (including the one in Eq. (14)), they reached the conclusion that for the two-measurement scenario the steering and Bell nonlocality are indistinguishable. Additionally, Quan *et al.*^74^ have demonstrated that the Bell-diagonal states are steerable iff they violate the CHSH inequality, considering two projective measurements also. What these works show is the existence of states in general (i.e. mixed) which are steerable and nonlocal at the same time; for example, those with CHSH-type correlations and the Bell-diagonal states.

That inequality, given by Eq. (14), adapted to the SGE takes the following form:

15 $$\begin{aligned} {\mathcal {B}}_z= & {} \sqrt{\left[ {\mathcal {C}}(z,\theta )+{\mathcal {C}}(z',\theta )\right] ^2+\left[ {\mathcal {C}}(z,\theta ')+{\mathcal {C}}(z',\theta ')\right] ^2}\nonumber \\&+\,\sqrt{\left[ {\mathcal {C}}(z,\theta )-{\mathcal {C}}(z',\theta )\right] ^2+\left[ {\mathcal {C}}(z,\theta ')-{\mathcal {C}}(z',\theta ')\right] ^2}\le 2 \end{aligned}$$

where

16 $$\begin{aligned} {\mathcal {C}}(z,\theta )=\langle {\Psi }| {\hat{W}}(z,p_z){\hat{\sigma }}(\theta )|{\Psi }\rangle , \end{aligned}$$

**Figure 3:**
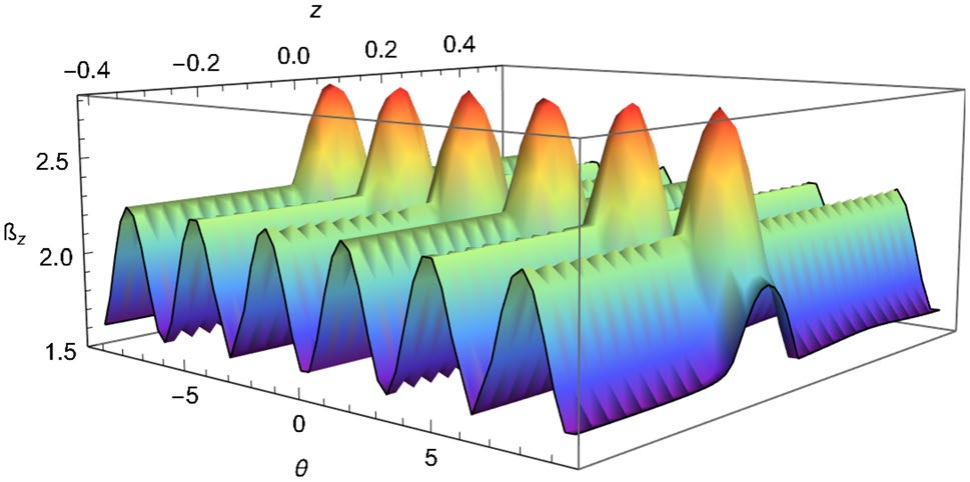
Plot of the inequality (15), taking $z' = 0.08$ and $\theta ' = \pi /2$, we have set $\pi \hbar =1$, $m=1$, $\sigma _0 =0.05$, $\mu cb / 2=2.2$, $p_z =0.01$ and time $t=0.2$.

**Figure 4:**
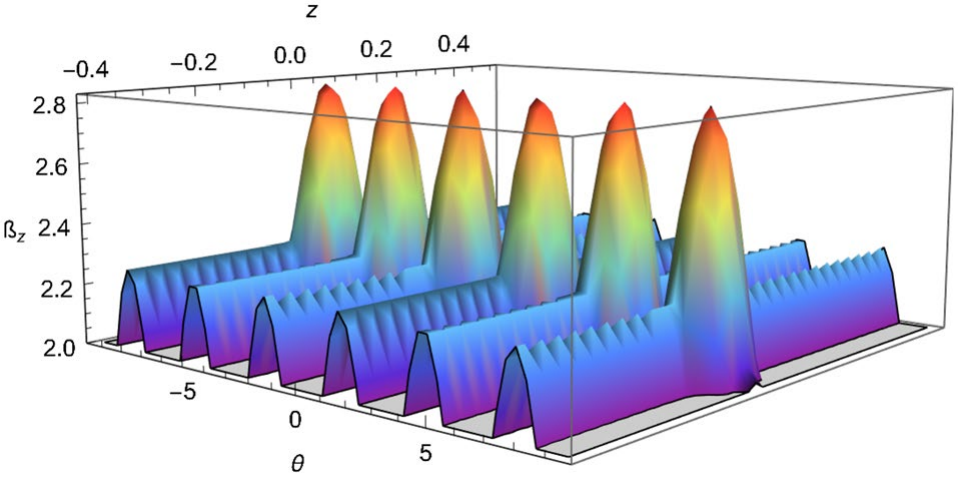
Inset of Fig. 3, for the values of ${\mathcal {B}}_z$ from 2 to 2.83.

**Figure 5:**
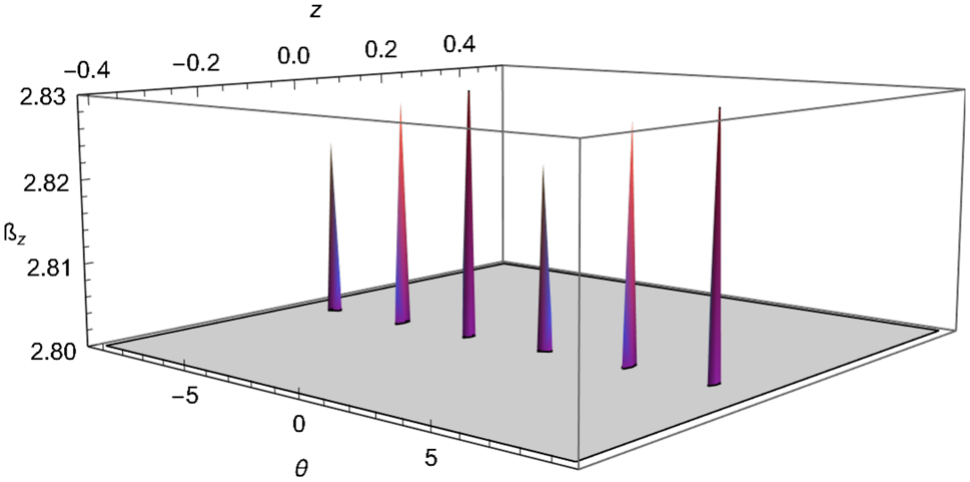
Inset of Fig. 3, for the values of ${\mathcal {B}}_z$ from 2.80 to 2.83.

${\hat{W}}(z,p_z)$ is the Wigner operator (we recall that the Wigner operator is equivalent to the parity operator^36^) and ${\hat{\sigma }}(\theta )$ is the Pauli operator in an arbitrary direction in $\theta$; see Eqs. (6) and (12) in reference^36^ where ${\mathcal {C}}(z,\theta )$ was calculated following the approach of Banaszek and Wódkiewicz^75,76^, please see reference^36^ for more details. A plot of ${\mathcal {B}}_z$, i.e. the inequality given in Eq. (15), is shown in Fig. 3. An inset of Fig. 3 is shown in Fig. 4, it shows the range of values of ${\mathcal {B}}_z$ between 2.0 and 2.83 which gives the violation of the steering inequality. Another inset of Fig. 3 is shown in Fig. 5, this shows how the correlation reaches the optimal value $2\sqrt{2}\approx 2.83$. Based on these plots, we certify that the SGE is capable of producing the steering phenomena.

## Methods

To certify steering we have followed two approach, the first one was by showing how the steering phenomena can be implemented by the wave function’s collapse by means of measuring suitable observables. The second one, was by showing the violation of an steering inequality, following the approach of Wódkiewicz^76^ who showed that by measuring the parity operator it can be demonstrated the nonlocality of quantum states, which translated to the SGE it shows that a LHS model can be constructed if the joint probability can be written as:

17 $$\begin{aligned} P(a,b|{\hat{W}}(z),{\hat{\sigma }}_z(\theta ),W)=\sum _\xi {\mathfrak {P}}(a|{\hat{W}}(z),\xi )P(b|{\hat{\sigma }}_z(\theta );\rho _\xi ){\mathfrak {P}}_\xi , \end{aligned}$$

where ${\hat{W}}(z)$ is the parity operator, i.e. the Wigner operator, and the dichotomic variables are ${\hat{W}}(z)$ and ${\hat{\sigma }}_z(\theta )$.

## Discussion and conclusion

Firstly, there is an unusual situation (a peculiar action-at-a-distance) coming from the above analysis: if Alice measured ${\hat{\sigma }}_z$ and detect nothing then the eigenfunction would collapse at Bob’s place towards $C_0^3M(x,y) \langle {z}|{\varphi _+}\rangle |{\uparrow _z}\rangle$; however, if Alice measured ${\hat{\sigma }}_x$ and detect $\hbar /2$ then the eigenfunction would collapse towards $C_0^5M(x,y) \left[ \langle {z}|{\varphi _+}\rangle +\langle {z}|{\varphi _-}\rangle \right] |{\uparrow _x}\rangle$, but if she detected $-\hbar /2$ the wave function would collapses towards $C_0^6M(x,y) \left[ \langle {z}|{\varphi _+}\rangle -\langle {z}|{\varphi _-}\rangle \right] |{\downarrow _x}\rangle$, remember that $\left[ \langle {z}|{\varphi _+}\rangle \pm \langle {z}|{\varphi _-}\rangle \right]$ is a superposition state of “being” in Paris and Tokyo at the same time, this fact demonstrates the truly nonlocal feature of the single-particle entanglement: How the particle senses which of the measuring devices Alice is using, i.e. the one that measures ${\hat{\sigma }}_z$ or the one that measures ${\hat{\sigma }}_x$? The answer is that the particle senses which device is in use by the nonlocality of the single-particle entanglement. This demonstrates that by measuring the internal degree of freedom, then, the external degree of freedom of the particle might be steered to become a reality in Paris or in Tokyo or even in a superposition state at both places.

Secondly, we can conclude that the spreading of the entangled wavefunction, of the single-particle entangled system, plays a paramount role in the nonlocality features that the system possesses. This open the question to whether such spreading plays a primordial role in the nonlocal properties of some of the multy-particle entangled systems.

Thirdly, the collapse of the wave function depending on what kind of measuring device Alice is using in Tokyo implies some similarity to the Young interferometer, in which the use of a measuring device near a pinhole plays a primordial role in the loss of the interference pattern. The difference lies in the fact that in the Young interferometer the presence of a measuring device rules out quantum effects whereas in the SGE the presence of a measuring device could be used to produce steering.

Fourthly, what the previous sections proved, by showing the violation of a steering inequality, is that Alice is capable to steer Bob state in Paris by choosing different measurements at Tokio. This is possible due to the spreading over large distances of the entangled wavefunction, i.e. the spreading of the single-particle entangled wavefunction. To produce the steering she ask Bob about the kind of state he wants, then after turning on the SGE by using a classical control protocol (capable of sending a single atom or *N* atoms, as she wish), she could be able to steer Bob quantum states in such a way that this process rules out the existence of local hidden state models^40^ (using some Bob’s protocol if he does not trust Alice). In concrete terms, by the use of *N* atoms Alice, by means of measuring the position observable, she will obtain *N*/2 times the eigenvalue $-z$ at Tokio, whereas Bob is going to obtain *N*/2 times the eigenvalue $+z$ at Paris. On the other hand, by the use of *N* atoms Alice, by means of measuring the spin ${\hat{\sigma }}_z$, she will obtain *N*/2 times the eigenvalue $-\hbar /2$ at Tokio, whereas Bob is going to obtain *N*/2 times the eigenvalue $+\hbar /2$ at Paris. Complex correlations could arise by measuring ${\hat{p}}_z$ or by measuring ${\hat{\sigma }}_x$.

Finally, these considerations provide strong support to the fact that single-particle entanglement together with the nonlocal features of its wavefunction can be used to produce nonlocal steering at two different faraway places, superseding contextuality.

However, further analysis and research must be undertaken to ascertain how the spin measurements and momentum measurements could be conducted independent of the position of the particle and further research must also be done to understand better the steering process when measuring two seemingly compatible observables when there is a hybrid entanglement^57^, for example *z* and ${\hat{\sigma }}_z$; specially for the case of bipartite entanglement with states of the form:

18 $$\begin{aligned} |{\psi (t)}\rangle= & {} \Big \{c_0 e^{-a_1\left( z_1+z_0(t)\right) ^{2}} e^{-a_2\left( z_2-z'_0(t)\right) ^{2}} |{\uparrow _z}\rangle _1|{\downarrow _z}\rangle _2 \nonumber \\&+\, c_1 e^{-a_1\left( z_1-z_0(t)\right) ^{2}} e^{-a_2\left( z_2+z'_0(t)\right) ^{2}} |{\downarrow _z}\rangle _1|{\uparrow _z}\rangle _2\Big \}, \end{aligned}$$

or similar ones, where the subscripts 1 and 2 refers to particle 1 and particle 2^1^.

## Footnotes

1 Just before this paper was approved for publication, I became aware of the paper by G. M. D’Ariano et al.^77^ where the authors propose a procedure for a quantum simultaneous measurement of the spin direction using an Arthurs–Kelly model,i.e. by simultaneous measurements of ${\hat{\sigma }}_x$, ${\hat{\sigma }}_y$ and ${\hat{\sigma }}_z$ without measuring the particle’s position. However, its implementation must still be analysed and checked further in order to asses its probable applicability to the SGE for the quantum measurement of the spin direction of ${\hat{\sigma }}_z$ only.
